# Functional Characterization of *PtoWOX1* in Regulating Leaf Morphogenesis and Photosynthesis in *Populus tomentosa*

**DOI:** 10.3390/plants14142138

**Published:** 2025-07-10

**Authors:** Feng Tang, Minghui He, Shi Liang, Meng Zhang, Xiaowei Guo, Yuxian Dou, Qin Song, Cunfeng Zhao, Ting Lan

**Affiliations:** 1 Key Laboratory of Eco-Environments of Three Gorges Reservoir Region, Ministry of Education, Chongqing Key Laboratory of Forest Resource Innovation and Utilization, Integrative Science Center of Germplasm Creation in Western China (Chongqing) Science City, School of Life Sciences, Southwest University, Chongqing 400715, China; tangfeng76@163.com (F.T.); heminghui1412@163.com (M.H.); liangshi@email.swu.edu.cn (S.L.); zhangmengerq_99@163.com (M.Z.); xiaowei.98@outlook.com (X.G.); 18955017573@163.com (Y.D.); songqin518@126.com (Q.S.); 2 Chongqing Institute of Green and Intelligent Technology, Chinese Academy of Sciences, Chongqing 400714, China; zhaocunfeng@cigit.ac.cn

**Keywords:** poplar, leaf morphogenesis, medio-lateral axis development, *PtoWOX1*

## Abstract

Leaves are essential for photosynthesis and transpiration, directly influencing plant growth and development. Leaf morphology, such as length, width, and area, affects photosynthetic efficiency and transpiration rates. In this study, we investigated the role of *PtoWOX1* in leaf morphogenesis by generating both overexpression and CRISPR/Cas9 knockout lines in *P. tomentosa*. The results showed that *PtoWOX1A* and *PtoWOX1B* encode nuclear-localized transcription factors highly expressed in young leaves, particularly in palisade and epidermal cells. Knockout of *PtoWOX1* resulted in reduced leaf width and area, enlarged upper epidermal cells, and lower stomatal density. Overexpression led to wrinkled leaf surfaces and reduced margin serration. Anatomical analysis revealed altered palisade cell arrangement and increased leaf thickness in knockout lines, accompanied by higher chlorophyll content and enhanced photosynthetic rates. Additionally, *PtoWOX1A* interacts with *PtoYAB3B*, suggesting a complex that regulates leaf margin development. These findings clarify the function of *PtoWOX1* in regulating mid-lateral axis development and leaf margin morphology and provide new insights for the molecular breeding of poplar.

## 1. Introduction

Plant leaves exhibit remarkable morphological diversity, shaped by both genetic programs and environmental cues. This plasticity enables plants to optimize light capture and photosynthetic efficiency under changing conditions [1,2]. Leaf development proceeds through three key stages: initiation of the leaf primordium, establishment of polarity, and laminar outgrowth [3,4,5]. A mature leaf exhibits three developmental axes: the proximal–distal axis, adaxial–abaxial axis, and the medio–lateral axis. The core regulatory network for leaf polarity includes members of the AS1/AS2, HD-ZIP III, KANADI (KAN), and YABBY (YAB) gene families [1,5,6,7].

Waites and Hudson proposed that the juxtaposition of adaxial and abaxial cell layers is essential for leaf blade outgrowth along the medio-lateral axis [8]. *WUSCHEL-related homeobox 1* (*WOX1*) is specifically expressed in the middle domain of the developing leaf and contributes to lamina expansion [9]. Similarly, Arabidopsis *WOX3* (also known as *PRESSED FLOWER*, *PRS*) is expressed at the adaxial–abaxial boundary at the leaf margin, playing roles in polarity maintenance and medial–lateral axis. While *wox1* or *prs* single mutants in Arabidopsis show no obvious phenotype, the *wox1prs* double mutants display narrow leaves, suggesting functional redundancy [10,11]. Functional studies in various species have shown that WOX1 and its homologs (*PRS* in *Arabidopsis*, *STF* in *Medicago*, *LAM1* in tobacco, and *MAW* in petunia) are required for normal laminar expansion. Mutants typically display narrow or radialized leaves, while their loss leads to disrupted leaf shape and vascular patterning [10,11,12]. In monocots, homologs such as *NAL* in rice and *NS* in maize similarly regulate laminar width [13,14,15], and in woody plants such as paper mulberry, *BpWOX1/3* mutations lead to deeper lobes and narrower leaflets [16], highlighting the functional conservation of WOX1/PRS across plant species.

Studies on *Medicago* show that STF can recruit the co-repressor TPL to form a transcriptional repression complex targeting *AS2*, a key adaxial regulator, to coordinate leaf margin development and medial-lateral growth [17]. *WOX1*/*PRS* expression in the middle domain is activated by *ARF5* (*MP*) on the adaxial side and repressed by *ARF2*/*4* on the abaxial side, implicating auxin signaling in leaf morphogenesis [18]. Disruption of polar auxin transport interferes with PIN1 convergence, misplaces SlLAM1 expression, and leads to radial symmetry and reduced medial-lateral leaf growth [19]. Recent research has indicated that WOX1/PRS may recruit other WOX family members to modulate leaf shape. The overexpression of *WOX9* in the *lam1* mutant enhances the phenotype, indicating antagonism between *WOX9* and *STF* during leaf development [20]. Furthermore, *STF* and *WOX9* directly regulate the expression of *CYTOKININ OXIDASE 3* (*CKX3*), affecting endogenous cytokinin levels and thus influencing cell proliferation and leaf growth [21]. These studies suggest that WOX1/PRS act as integrators of auxin and cytokinin signaling to promote cell division and lamina expansion. Mechanical studies in Arabidopsis further support this model: anisotropic cell wall constraints, such as cellulose microfibril orientation along the adaxial–abaxial axis, create higher tension in this direction, forcing cells to expand and divide along the medial–lateral axis [21,22]. This implies that medial–lateral growth depends on proper proximal–distal axis establishment.

Despite extensive research in herbaceous models, studies on leaf development in woody species remain limited. Poplar is a fast-growing, ecologically important tree with high economic value and significant leaf shape diversity [23,24,25], making it an ideal model for exploring leaf morphogenesis in trees. To better understand the biological role of *WOX1* in woody plants, we conducted a functional analysis of *PtoWOX1* in *P. tomentosa*. We generated transgenic poplar lines with altered *PtoWOX1* expression through Agrobacterium-mediated transformation and conducted detailed phenotypic and cytological analyses to investigate changes in leaf morphology and cell size. This research aims to elucidate the biological function of *PtoWOX1* in leaf development and contribute to a deeper understanding of the genetic regulation of leaf morphogenesis in woody plants. Moreover, our findings will offer valuable insights for the molecular breeding of poplar and other trees with improved growth, stress tolerance, and ornamental value.

## 2. Results

### 2.1. Identification of PtoWOX1 in P. tomentosa

**Figure 1:**
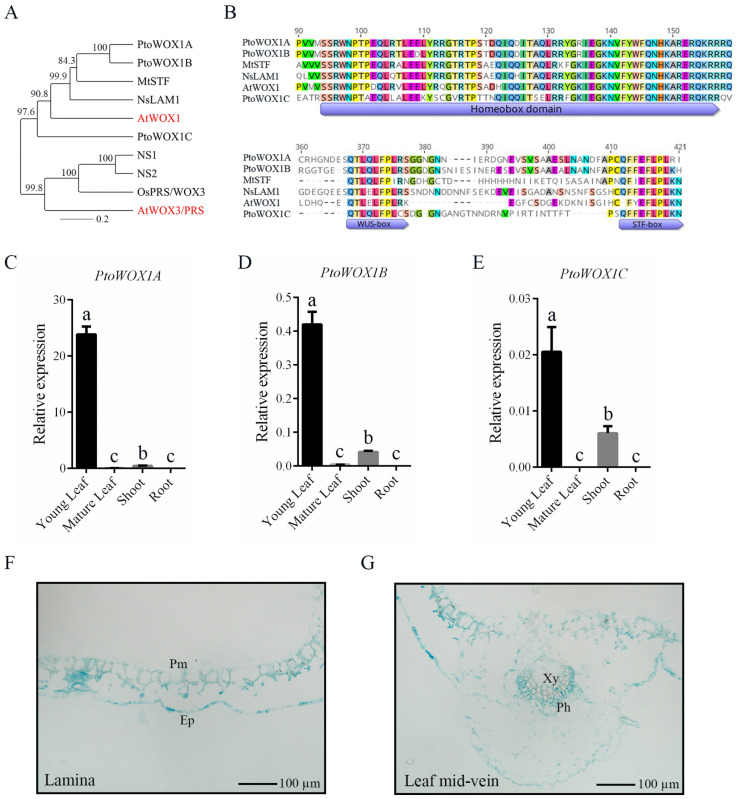
Phylogenetic analysis and expression patterns of *PtoWOX1* in *P. tomentosa.* (**A**) Phylogenetic analysis of WOX1 proteins from *P. tomentosa*, *Arabidopsis thaliana*, *Medicago truncatula*, tobacco, and rice. (**B**) Amino acid sequence alignment of *PtoWOX1* and its homologs, with conserved domains including the Homeobox, WUS-box, and STF-box highlighted by purple boxes. (**C**–**E**) Expression pattern of *PtoWOX1A* (**C**), *PtoWOX1B* (**D**), and *PtoWOX1C* (**E**) in roots, stems, and leaves of *P. tomentosa*. The poplar *Ubiquitin* gene (*UBQ*) was used as the internal reference gene. The expression levels of *PtoWOX1* were normalized to the *UBQ* gene and calculated using the 2^−ΔΔCt^ method. Data are presented as means ± standard deviation (SD), *n* = 3 biological replicates. Significant differences were tested using one-way ANOVA followed by Tukey’s test: different letters represent significant differences. (**F**,**G**) Expression of *PtoWOX1A* in leaf *lamina* (**F**) and leaf mid-veins (**G**) of *P. tomentosa* by GUS staining. Ep: epidermis; Pm: palisade mesophyll; Xy: xylem; Ph: phloem.

Using the coding sequence (CDS) of *Arabidopsis thaliana WOX1/PRS*, a BLAST (Geneious Prime version 2019.2.1) search was conducted against the *P. tomentosa* genome database. Three *PtoWOX1* genes were identified based on sequence alignment and phylogenetic analysis, named *PtoWOX1A* (*P.x_tomentosa74822.t1*), *PtoWOX1B* (*P.x_tomentosa70482.t1*), and *PtoWOX1C* (*P.x_tomentosa22998.t1*). Phylogenetic analysis of WOX1/PRS members revealed two clades: the WOX1 and WOX3 sub-clades. All three *PtoWOX1* members belonged to the WOX1 sub-clade. Among them, *PtoWOX1A* and *PtoWOX1B* were closely related to *AtWOX1*, *STF*, and *LAM1* (Figure 1A). Multiple sequence alignment of WOX1 homologs showed that *PtoWOX1A*, *PtoWOX1B*, and *PtoWOX1C* all contained the conserved Homeobox Domain (HD), WUS-box, and STF-box motifs (Figure 1B).

The expression patterns of *PtoWOX1A*, *PtoWOX1B*, and *PtoWOX1C* were examined independently. *PtoWOX1A* exhibited strong tissue-specific expression, with predominant accumulation in young leaves and negligible levels in other tissues (Figure 1C). *PtoWOX1B* also showed its highest expression in young leaves, although expression was observed in shoots as well (Figure 1D). In contrast, *PtoWOX1C* was expressed at much lower levels, with weak signals detected in young leaves and shoots (Figure 1E). These results indicate that *PtoWOX1A* is likely the primary functional gene regulating leaf development in poplar. To characterize the expression pattern of *PtoWOX1A* in leaves, a GUS reporter gene driven by the *PtoWOX1A* promoter was constructed and transformed into wild-type *P. tomentosa*. The results showed that *PtoWOX1A* was predominantly expressed in palisade mesophyll and epidermal cells (Figure 1F). GUS staining signals were also detected in vascular tissues, specifically in the phloem and mesophyll cells of the main leaf vein (Figure 1G). The subcellular localization assays showed that both *PtoWOX1A* and *PtoWOX1B* were localized to the nucleus (Supplementary Figure S1), supporting their roles as transcription factors. Given the low expression of *PtoWOX1C* in leaves and its distant phylogenetic relationship with *STF* and *LAM*, *PtoWOX1A* and *PtoWOX1B* were selected as the primary targets for further investigation.

### 2.2. PtoWOX1 Regulates Leaf Morphogenesis in P. tomentosa

To investigate the role of *PtoWOX1* in leaf development, the CDS of *PtoWOX1A* and *PtoWOX1B* were amplified from leaf cDNA and cloned into the pCXSN vector. The constructs were introduced into *P. tomentosa*, and three independent overexpression lines were generated for each gene. Quantitative PCR analysis showed that *PtoWOX1A* was overexpressed more than 30-fold in lines 10, 12, and 15 (Supplementary Figure S2A), while *PtoWOX1B* was overexpressed over 500-fold in lines 2, 3, and 4 (Supplementary Figure S2B). After two weeks of growth in soil, both *PtoWOX1A* and *PtoWOX1B* overexpression plants (hereafter referred to as *PtoWOX1A*-OE and *PtoWOX1B*-OE) displayed dwarf phenotypes with wrinkled leaf surfaces and reduced leaf size compared to the wild type (Supplementary Figure S2C). In addition, we obtained the double-knockout mutants for *PtoWOX1A* and *PtoWOX1B* (hereafter referred to as *PtoWOX1* knockout lines, *PtoWOX1*-KO) through the CRISPR/Cas9 system. Sequencing analysis revealed a 31 bp deletion of *PtoWOX1A* and a 1-bp deletion of *PtoWOX1B* in line 3, while line 2 showed a 1 bp insertion in both *PtoWOX1A* and *PtoWOX1B* (Supplementary Figure S3A,B). After two weeks of growth in soil, line 2 exhibited a filamentous leaf phenotype reminiscent of the *lam1* mutant in tobacco, whereas line 3 showed a narrow leaf phenotype similar to the *stf* mutant in Medicago (Supplementary Figure S3C).

**Figure 2:**
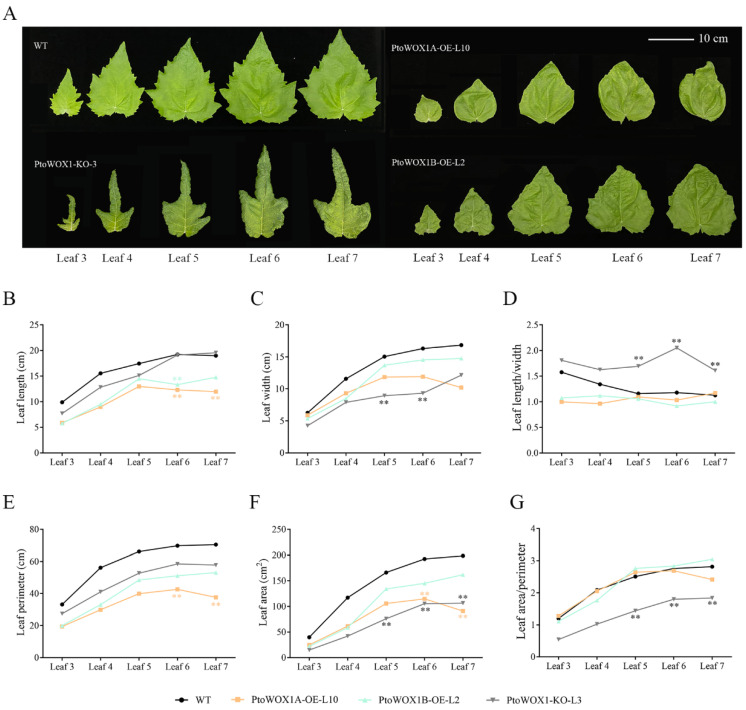
Phenotypes of *P. tomentosa PtoWOX1* transgenic line leaves. (**A**) Leaf morphology of *PtoWOX1* transgenic lines after two months of soil cultivation. “Leaf 3” indicates the third leaf from the shoot apex, and so on. Scale bar = 10 cm. (**B**–**G**) Measurement of leaf length (**B**), leaf width (**C**), leaf length-to-width ratio (**D**), leaf area (**E**), leaf perimeter (**F**), and leaf area-to-perimeter ratio (**G**) in *PtoWOX1* transgenic lines. Data are presented as means ± standard deviation (SD), *n* = 3 biological replicates. Student’s *t*-test: *p* < 0.01 (**).

Measurements of leaf length, width, area, and perimeter in *PtoWOX1* transgenic lines were performed after two months of growth. Compared to the wild type (WT), *PtoWOX1A*-OE and *PtoWOX1B*-OE lines displayed slightly reduced leaf length and width, while *PtoWOX1B*-KO lines showed a significant reduction in leaf width (Figure 2A–C). The leaf length-to-width ratio (L/W) of young leaves in WT was around 1.5, decreasing to ~1.1 in mature leaves (sixth and seventh leaves). In contrast, the L/W ratios were slightly lower in the overexpression lines and significantly higher in the knockout lines compared to WT, primarily due to reduced leaf width in the knockout lines (Figure 2D). These results suggest that *PtoWOX1* may regulate leaf growth along the medio-lateral axis. Leaf area and perimeter were both reduced in *PtoWOX1* transgenic lines compared to WT (Figure 2E,F). However, due to the significant reduction in area, the area-to-perimeter ratio (A/P) of the knockout lines was higher than that of WT. In contrast, no obvious difference in A/P ratio was observed in the overexpression lines (Figure 2G). These findings suggest that *PtoWOX1* may also be involved in regulating leaf margin morphology.

### 2.3. PtoWOX1 Influences Epidermal Cell Development in P. tomentosa

**Figure 3:**
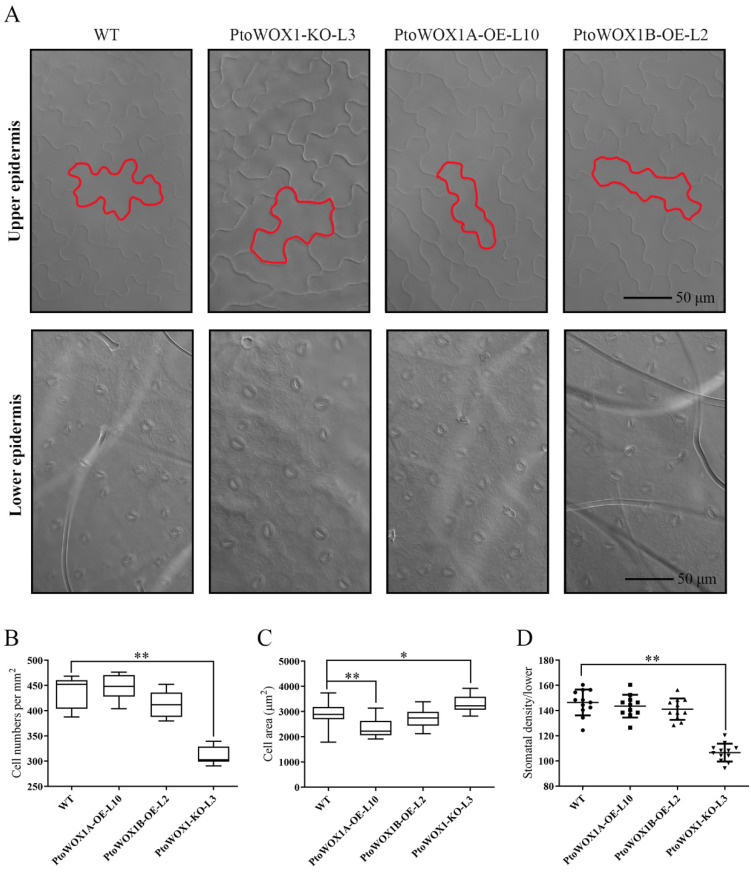
Epidermal cell morphology of *PtoWOX1* transgenic lines in *P. tomentosa*. (**A**) Epidermal cell morphology in *PtoWOX1* transgenic lines. Scale bar = 50 µm. (**B**) Number of upper epidermal cells per mm^2^ (cells/mm^2^). Student’s *t*-test: *p* < 0.01 (**). (**C**) Area of upper epidermal cells (µm^2^). Student’s *t*-test: *p* < 0.05 (*), *p* < 0.01 (**). (**D**) Stomatal density in the lower epidermis (stomata/mm^2^). Data are presented as means ± standard deviation (SD), *n* = 3 biological replicates. Student’s *t*-test: *p* < 0.01 (**).

Previous studies have shown that the morphology of leaf epidermal cells can influence leaf shape. To examine this, we analyzed the epidermal cell characteristics in *PtoWOX1* transgenic lines (Figure 3A). In the *PtoWOX1*-KO lines, epidermal cell size was significantly increased, while cell number per unit area was markedly reduced. In contrast, *PtoWOX1A*-OE lines exhibited a notable reduction in epidermal cell size with no significant change in cell number. Meanwhile, *PtoWOX1B*-OE had no evident effect on either cell size or number (Figure 3B,C). These findings suggest that *PtoWOX1* may regulate leaf morphology by modulating epidermal cell size and/or proliferation. In addition, we analyzed stomatal density on the abaxial surface of leaves from the transgenic lines. While no significant changes were observed in *PtoWOX1A*-OE and *PtoWOX1B*-OE lines, stomatal density was significantly reduced in the *PtoWOX1*-KO line (Figure 3A,D). Given the role of stomata in gas exchange during photosynthesis, these results indicate that *PtoWOX1*-mediated changes in leaf morphology may also influence photosynthetic efficiency.

### 2.4. PtoWOX1 Influences Photosynthetic Efficiency in P. tomentosa

**Figure 4:**
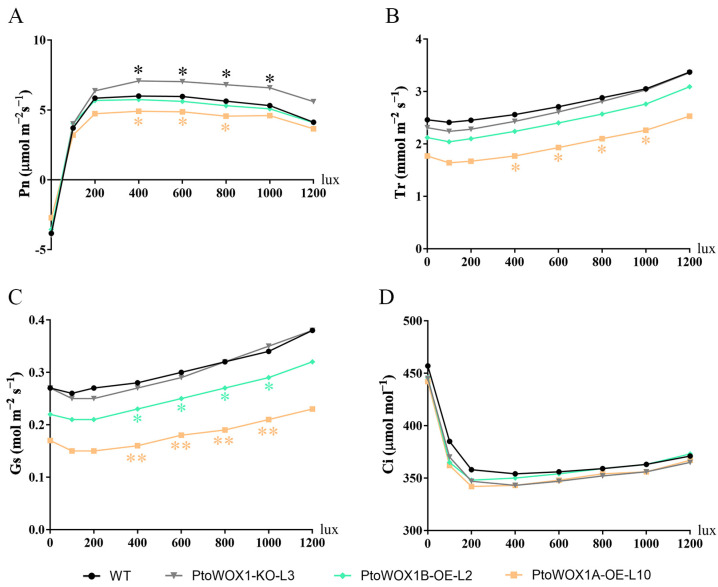
Photosynthetic parameters of *PtoWOX1* transgenic lines in *P. tomentosa*. Photosynthetic parameter measurement of net photosynthetic rate, Pn (**A**); transpiration rate, Tr (**B**); stomatal conductance, Gs (**C**); and intercellular CO_2_ concentration, Ci (**D**) in *PtoWOX1* transgenic lines. Data are presented as means ± standard deviation (SD), *n* = 2 biological replicates. Student’s *t*-test: *p* < 0.05 (*), *p* < 0.01 (**).

Photosynthesis is fundamental to plant growth and development, and variations in leaf morphology can affect photosynthesis. Previous studies have shown a positive correlation between chlorophyll content and photosynthetic efficiency, with higher chlorophyll levels generally associated with enhanced photosynthetic capacity and greater adaptability. To investigate the role of *PtoWOX1* in photosynthesis, we measured the total chlorophyll content in *PtoWOX1* transgenic lines. The results showed that chlorophyll content was higher in *PtoWOX1*-KO lines, whereas it was slightly lower in the overexpression lines (Supplementary Figure S4). These findings suggest that *PtoWOX1* may influence photosynthetic capacity. To further test this hypothesis, we assessed photosynthetic parameters using a portable LCi T LCpro system. The measurements included the net photosynthetic rate (Pn), the transpiration rate (Tr), stomatal conductance (Gs), and the intercellular CO_2_ concentration (Ci). Compared with WT leaves, the *PtoWOX1*-KO lines exhibited a significantly increased net photosynthetic rate (Figure 4A), while Tr, Gs, and Ci remained largely unchanged (Figure 4B–D). Interestingly, despite the significant enhancement in photosynthetic efficiency, the plant height of *PtoWOX1*-KO lines did not show a statistically significant difference compared to WT plants (Figure S3). This suggests that increased photosynthetic activity did not directly translate into vertical growth under current conditions. In contrast, *PtoWOX1A* and *PtoWOX1B* overexpression lines displayed reduced Pn, Tr, and Gs, with no significant changes in Ci (Figure 4A–D). These results indicate that *PtoWOX1* negatively regulates photosynthetic efficiency in *P. tomentosa*, and the narrow-leaf phenotype may represent an “ideal leaf shape”.

### 2.5. PtoWOX1 Affects Palisade Cell Morphology in P. tomentosa

**Figure 5:**
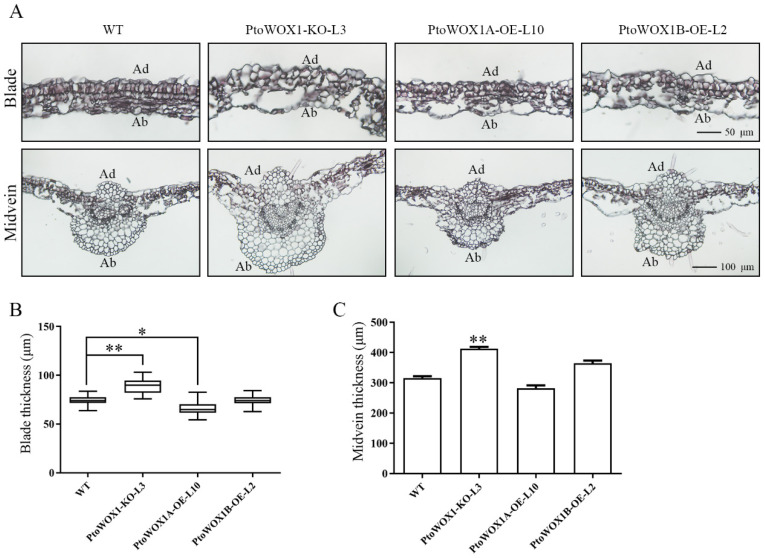
Longitudinal sections of leaf in *PtoWOX1* transgenic lines. (**A**) Cellular morphology of longitudinal leaf sections in *PtoWOX1* transgenic lines. Scale bar = 50 µm for mesophyll cells, 100 µm for midvein cells. Ad: adaxial; Ab: abaxial. (**B**,**C**) Thickness of mesophyll cells (**B**) and leaf midvein (**C**). Data are presented as means ± standard deviation (SD), *n* = 3 biological replicates. Student’s *t*-test: *p* < 0.05 (*), *p* < 0.01 (**).

To further elucidate the role of *PtoWOX1* in leaf development, we conducted longitudinal sectioning of the sixth leaf in *PtoWOX1* transgenic lines. In WT, a distinct two-layered palisade mesophyll structure was observed, characterized by regular shapes and tightly packed arrangements. In contrast, the *PtoWOX1*-KO line displayed irregularly shaped and disorganized palisade cells. Although *PtoWOX1A* and *PtoWOX1B* overexpression lines retained relatively intact palisade structures, the cells were less regular and compact compared to WT (Figure 5A). Measurements of leaf and midvein thickness further supported these observations. The knockout lines exhibited significantly increased leaf thickness compared to WT. In the *PtoWOX1A*-OE line, mesophyll thickness was slightly reduced, with no significant difference observed in midvein thickness. No changes in either mesophyll or midvein thickness were found in the *PtoWOX1B*-OE line (Figure 5B,C). To better understand the impact of *PtoWOX1*-KO L2 on leaf polarity, we performed histological analyses on the L2 mutant line (filamentous leaf). The results revealed a complete absence of palisade tissue in the leaf structure (Supplementary Figure S4A), indicating a disruption in adaxial–abaxial polarity. However, the polarity of vascular tissues, including xylem and phloem, remained intact (Supplementary Figure S4B). These findings suggest that *PtoWOX1* plays a crucial role in establishing adaxial–abaxial polarity during leaf development in *P. tomentosa*.

### 2.6. PtoWOX1 and PtoYAB3B Cooperatively Regulate Leaf Morphogenesis in P. tomentosa

**Figure 6:**
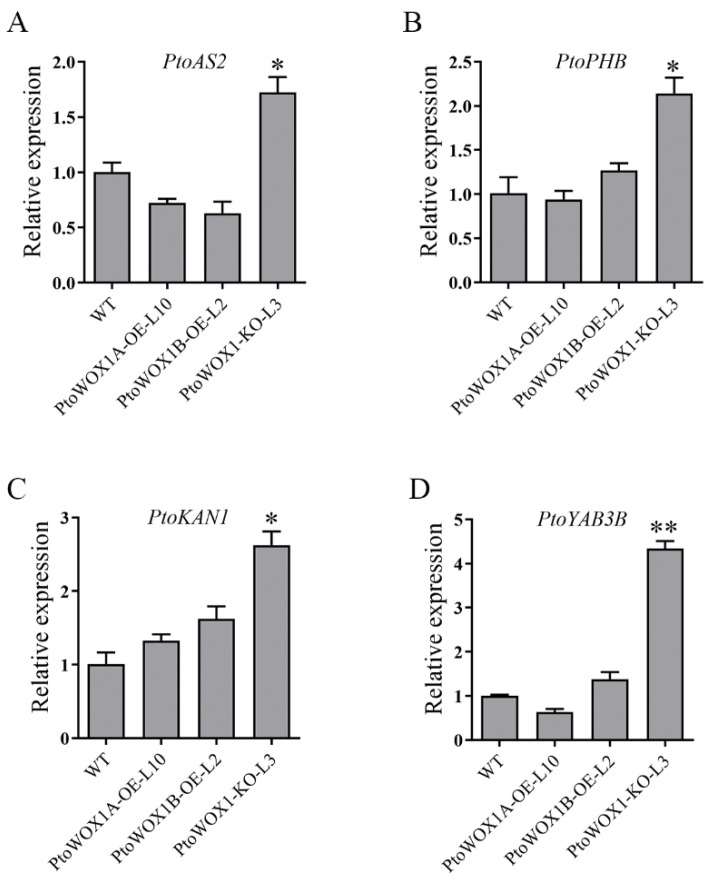
Expression analysis of leaf polarity regulators in *PtoWOX1* transgenic lines of *P. tomentosa*. (**A**–**D**) Expression of the leaf development genes *PtoAS2* (**A**), *PtoPHB* (**B**), *PtoKAN1* (**C**), and *PtoYAB3B* (**D**) in *PtoWOX1* transgenic lines. The poplar *UBQ* was used as the internal reference gene. Data are presented as means ± standard deviation (SD), *n* = 3 biological replicates. Student’s *t*-test: *p* < 0.05 (*), *p* < 0.01 (**).

Previous studies have demonstrated that genes involved in adaxial–abaxial polarity play crucial roles in leaf morphogenesis, and the altered expression of these genes can lead to distinct leaf shapes [5,26]. To explore the regulatory role of *PtoWOX1* in leaf polarity, we examined the expression levels of key adaxial–abaxial-associated genes. The expression levels of all tested polarity genes—including the adaxial markers *PtoAS2* and *PtoPHB* and the abaxial markers *PtoKAN1* and *PtoYAB3B*—were significantly upregulated in the *PtoWOX1-KO* lines (Figure 6A–D). In contrast, *PtoAS2* was notably downregulated in both *PtoWOX1A-OE* and *PtoWOX1B-OE* lines, while *PtoYAB3B* showed a slight decrease only in the *PtoWOX1A-OE* line and remained slightly increased in the *PtoWOX1B-OE* line. These expression patterns suggest that *PtoWOX1* may negatively regulate leaf polarity genes, particularly *PtoAS2* and *PtoYAB3B*. Previous work has identified *PtoYAB11* (*PtoYAB2C*) and *PtoYAB4* (*PtoYAB3B*) as regulators of leaf margin development in poplar. *PtoYAB4* promotes the expression of *PtoNGAL1*, which in turn represses *PtoCUC2*, resulting in a phenotype with reduced serration at the leaf margin [27]. To investigate whether *PtoWOX1* interacts with the PtoYAB, we constructed AD-*PtoWOX1* and BD-*PtoYAB* fusion vectors and co-transformed them into *Saccharomyces cerevisiae* strain AH109. Yeast two-hybrid assays revealed that both *PtoWOX1A* and *PtoWOX1B* interact with *PtoYAB3* and *PtoFIL* proteins (Supplementary Figure S6), suggesting that the reduced leaf serration observed in *PtoWOX1*-OE lines may be associated with the *PtoYAB* gene.

**Figure 7:**
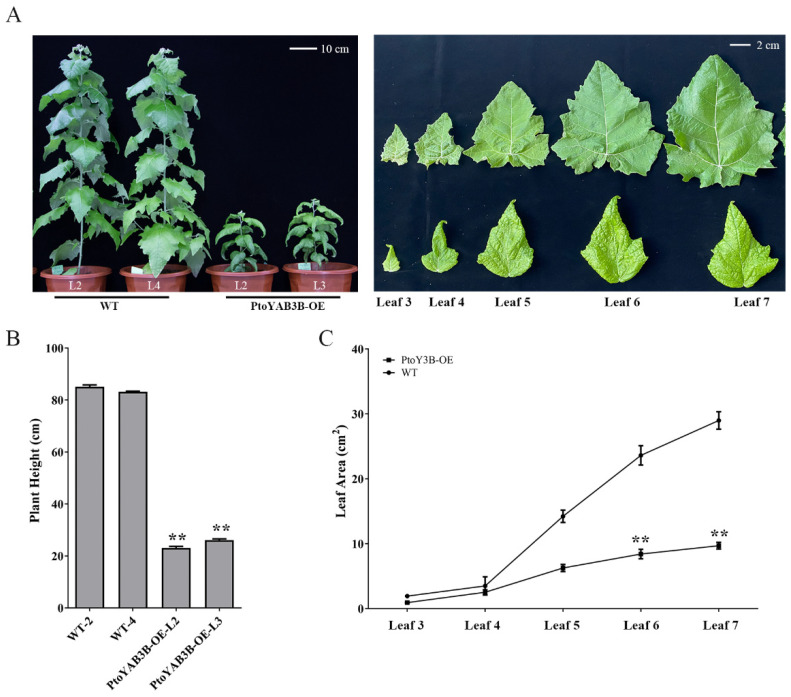
Phenotypic characterization of *PtoYAB3B* overexpression lines in *P. tomentosa*. (**A**) Phenotypes of *PtoYAB3B-OE* transgenic plants after three months of soil cultivation. Scale bar = 10 cm (**left**), 2 cm (**right**). (**B**) Plant height in *PtoYAB3B-OE* transgenic lines. Student’s *t*-test: *p* < 0.01 (**). (**C**) Leaf area in *PtoYAB3B-OE* transgenic lines. Data are presented as means ± standard deviation (SD), *n* = 3 biological replicates. Student’s *t*-test: *p* < 0.01 (**).

To further validate the role of *PtoYAB3B*, we analyzed the leaf phenotypes of its overexpression and knockout lines. Only *PtoYAB3B* overexpression lines exhibited reduced serration along the leaf margin, similar to the phenotype observed in *PtoWOX1* overexpression lines. These leaves also displayed wrinkled surfaces and abaxial curling at the margins (Figure 7A). Additionally, the *PtoYAB3B* overexpression lines exhibited a significant reduction in plant height (Figure 7B) and a marked decrease in leaf area (Figure 7C). These results suggest that *PtoWOX1* and *PtoYAB3B* may cooperatively regulate the leaf margin morphogenesis in *P. tomentosa*.

## 3. Discussion

### 3.1. PtoWOX1 Is Involved in the Mid-Lateral Growth of Leaves in P. tomentosa

The WUSCHEL-related homeobox (WOX) gene family comprises a group of plant-specific transcription factors that play crucial roles in the proliferation and differentiation of stem cell niches, including the shoot apical meristem, root apical meristem, and vascular cambium. Additionally, WOX genes are involved in lateral organ development, organ size determination, and vascular tissue differentiation [28]. In both monocotyledonous and dicotyledonous species such as Arabidopsis, Medicago, tobacco, rice, and maize, loss-of-function of WOX1/3 homologs consistently exhibit a reduction in leaf blade width [10,12,13,15]; for instance, a narrow-leaf phenotype was observed in Medicago and a filamentous leaf resulting from the complete absence of mesophyll cells in tobacco [9,12]. These genes predominantly influence leaf development by regulating cell enlargement and cell proliferation [21]. Interestingly, in monocotyledonous lineages, the WOX1 clade appears to be absent. Instead, maize *NS1/NS2* and rice *NAL2/3* are more closely related to AtWOX3/PRS, whereas *Medicago STF* and tobacco *LAM* are closer to AtWOX1. In *P. tomentosa*, both *PtoWOX1A* and *PtoWOX1B* are closely related to STF and LAM within the WOX1 clade (Figure 1A). Knockout lines of *PtoWOX1* exhibit narrow-leaf and filamentous-leaf morphologies resembling those of *stf* and *lam* mutants (Figure S3C). Similarly, in the woody species *Broussonetia papyrifera*, double mutants of *BpWOX1* and *BpWOX3* also show a significant reduction in leaf width [16]. These findings indicate that the function of WOX1 in regulating leaf width is evolutionarily conserved, even in woody plants. Collectively, these observations suggest that WOX1/3 genes have retained conserved functions during evolution, particularly in promoting medio-lateral leaf growth.

Previous studies have demonstrated that modern clade WOX genes containing the WUS-box domain (e.g., WUS, WOX1-WOX6) can partially or fully rescue the narrow-leaf phenotype observed in *stf*/*lam1* mutants [9]. Moreover, functional interchangeability has been reported between *WOX5* (expressed in the root apical meristem) and *WUS* (expressed in the shoot apical meristem), suggesting a high degree of functional conservation among the modern WOX clade members [29]. The involvement of WOX1 in medio-lateral leaf growth is closely associated with the presence of the C-terminal WUS-box domain [9]. Consistent with these findings, we found that the *PtoWOX1*-KO plant also shows a narrow-leaf phenotype, due to the loss of the WUS-box domain. In contrast, ectopic expression of the intermediate clade member *WOX9* in *lam1/stf* mutants exacerbates the mutant phenotype, whereas antisense suppression of *WOX9* partially rescues it. This antagonistic interaction between *WOX9* and *WOX1* indicates a coordinated regulatory mechanism in controlling leaf blade flattening [20,21]. These results highlight the critical role of the WUS-box in medio-lateral leaf development.

### 3.2. PtoWOX1 and PtoYAB3B Synergistically Regulate Leaf Margins

WOX1 is also involved in the morphogenesis of leaf margins [17]. Previous studies have shown that *PtoYAB11* harbors a premature termination codon (designated *PtoYAB11^^PSC^*), which leads to the loss of the zinc finger domain and consequently abolishes its ability to transcriptionally activate the downstream target gene *PtoNGAL-1*. This results in the accumulation of *PtoCUC*, a downstream gene of *PtoNGAL-1*, ultimately enhancing serration development at the leaf margin [27]. In *P. tomentosa*, the overexpression of *PtoWOX1A* and *PtoWOX1B* resulted in wrinkled and uneven leaf surfaces, accompanied by a reduction in marginal serration (Figure 2A). In *Arabidopsis*, *WOX1* functions downstream of *YAB* genes during leaf polarity development, whereas in rice, *OsWOX3* directly regulates *OsYAB3* during shoot apex formation. These results suggest a potential interaction between *PtoWOX1* and *YABBY* genes (*PtoYAB*) in regulating leaf margin morphology. In *Populus*, QTL and GWAS analyses of leaf shape traits revealed that *PtrWOX1B* and *PtrYAB* genes are co-expressed within the same gene regulatory network [23,30,31,32], suggesting that *PtoWOX1* and *PtoYAB* may function within a shared regulatory pathway during leaf margin development. Yeast two-hybrid (Y2H) assays demonstrated that both *PtoWOX1A* and *PtoWOX1B* could physically interact with four members of the *PtoYAB3/FIL* subfamily (Figure S6). Although the Y2H assay indicated a potential interaction between *PtoWOX1* and *PtoYAB3*, this result should be interpreted with caution. Further validation using bimolecular fluorescence complementation (BiFC) or other in vivo interaction assays is necessary to confirm their physical interaction in plants. Morphological analysis of overexpressed lines in *PtoYAB3B* (*PtoYAB4*) showed a noticeable reduction in leaf margin serration (Figure 7). *PtoYAB4* functions as a positive regulator of *PtoNGAL1* [27]. These results indicate that *PtoWOX1* may form a protein complex with *PtoYAB3B*, jointly regulating the expression of downstream pathway genes such as *PtoNGAL* and *PtoCUC2*, thereby participating in the morphogenesis of the leaf margin.

### 3.3. Narrow-Leaf Phenotype Is an ‘Ideal Leaf Shape’

Once leaf polarity is properly established, the leaf morphology is determined by its flattening growth [33]. Variations in leaf shape influence a plant’s adaptability to the environment. The narrow-leaf phenotype presents several advantages under the concept of the ‘ideal leaf shape.’ For instance, in the genus *Isodon*, deeply lobed (narrow) leaves confer resistance to insect herbivory. The weevil preferentially inhabits the non-lobed *I. trichocarpus*, while the lobed *I. umbrosus* exhibits reduced susceptibility to damage [34]. In upland cotton (*Gossypium hirsutum*), the okra leaf type, characterized by deep incisions and narrow lobes, allows better air circulation and light penetration, creating unfavorable conditions for pests. The okra leaf type offers advantages in upland cotton breeding programs [35,36]. In this study, the narrow-leaf phenotype observed in *PtoWOX1* knockout lines exhibited significantly enhanced photosynthetic efficiency (Figure 4), demonstrating the potential of gene editing to improve photosynthetic capacity and offering new perspectives for molecular breeding in trees.

## 4. Materials and Methods

### 4.1. Plant Materials and Growth Conditions

*P. tomentosa* was cultivated in a greenhouse under a 16 h light/8 h dark cycle, with the light period maintained at 25 °C and a photosynthetic photon flux density of 5000 lux, and the dark period at 23 °C. The relative humidity was consistently maintained at 60% throughout the cultivation period.

### 4.2. Vector Construction and Plant Transformation

The full-length cDNA of the *PtoWOX1A* and *PtoWOX1B* genes was amplified from *P. tomentosa* cDNA using gene-specific primers (Table S1) and cloned into a pCXSN vector. The construct was stably transformed to wild-type *P. tomentosa* plants through the method of *Agrobacterium*-mediated infiltration of leaf disks, as described previously [37]. Positive transgenic lines were identified via PCR with gene-specific primers and subsequent kanamycin or hygromycin resistance selection.

### 4.3. RNA Extraction and Quantitative RT-PCR

Total RNA was extracted from transgenic plant stems using the Biospin Plant Total RNA Extraction Kit (Bioflux, Hangzhou, China). Total RNA was reverse-transcribed into cDNA using the PrimeScript™ RT Reagent Kit with the gDNA Eraser (TaKaRa, Dalian, China). Real-time quantitative PCR (RT-qPCR) was performed using SYBR^®^ Premix Ex Taq™ (TaKaRa) on a qTOWER^3^ G IVD real-time PCR system (Analytik Jena AG, Jena, Germany). The poplar *UBQ* (*P.x_tomentosa49626.t1*) was used as the internal reference gene for RT-qPCR analysis. Expression levels in Figure 1C–E were normalized to the *UBQ* gene. The primers used for RT-qPCR are listed in Table S1. The relative expression levels of target genes were calculated using the 2^−ΔΔCt^ method. Three biological and three technical replicates were performed for each gene to ensure reproducibility.

### 4.4. Paraffin Sectioning of Leaves

Leaf samples were fixed in FAA solution (formalin–acetic acid–50% ethanol = 5:5:90, *v*/*v*/*v*) overnight at 4 °C, dehydrated through a graded ethanol series, cleared in xylene, and embedded in paraffin wax (melting point 60 °C). Sections (8–10 μm thick) were prepared using a rotary microtome and mounted on poly-L-lysine-coated slides. For histological staining, sections were dewaxed, rehydrated, and stained with 1% safranin O for 4–6 h, then counterstained with 0.5% fast green for 5–30 s. After dehydration and clearing, slides were sealed with neutral resin and observed using a microscope (Olympus BX53, Olympus Corporation, Tokyo, Japan).

### 4.5. GUS Staining

The positive transgenic lines containing the *GUS* reporter gene driven by the *PtoWOX1A* promoter were identified. The cross-section of leaves underwent fixation in acetone for one hour at 20 °C, followed by two wash cycles in double-distilled H_2_O (ddH_2_O). Subsequently, the samples were incubated in staining buffer in the dark at 37 °C for 3 h. The staining buffer contained 50 mM sodium phosphate buffer (pH 7.0), 2 mM X-Gluc (5-bromo-4-chloro-3-indolyl β-D-glucuronide), 0.5 mM potassium ferricyanide, 0.5 mM potassium ferrocyanide, 10 mM EDTA, and 0.1% (*v*/*v*) Triton X-100. Chlorophyll extraction was performed using a destaining solution (ethanol–acetic acid, 3:1 ratio) at room temperature for 30 min, followed by two ddH_2_O rinses. The chlorophyll-free stained materials were then subjected to paraffin sectioning and examined. Digital images were captured using an Olympus BX53 microscope.

### 4.6. Leaf Epidermis Observation

Fresh poplar leaves were cut into 3–5 mm square pieces, avoiding major veins. The samples were incubated in HCG solution (100 mL: 80 g chloral hydrate, 10 mL glycerol, 30 mL ddH_2_O) overnight at room temperature. If clearing was insufficient, the solution was replaced with fresh HCG and the incubation was continued until adequate transparency was achieved. Cleared samples were then observed under a differential interference contrast (DIC) microscope to examine epidermal cells.

### 4.7. Measurement of Photosynthetic Parameters

Leaves from the same position on WT and PtoWOX1 transgenic lines were selected for measurements. A portable photosynthesis system (LCi T LCpro, ADC BioScientific Ltd., Hoddesdon, UK) was used to measure the light response curve under a series of photosynthetically active radiation (PAR) intensities: 0, 100, 200, 400, 600, 800, 1000, and 1200 µmol m^−2^s^−1^. The measured parameters included the net photosynthetic rate (Pn), the transpiration rate (Tr), stomatal conductance (Gs), and the intercellular CO_2_ concentration (Ci). For each genotype, three biological replicates (individual trees) were measured, and the average values were calculated for analysis.

### 4.8. Subcellular Localization Assay

The full-length coding sequence of *PtoWOX1* (without the stop codon) was amplified and cloned into a modified pCAMBIA1300 vector carrying a *GFP* reporter gene under the control of the CaMV 35S promoter. The recombinant plasmid (35S::PtoWOX1-GFP) and the control vector (35S::GFP) were introduced into *Agrobacterium tumefaciens* strain GV3101. The *Agrobacterium* cultures were infiltrated into *Nicotiana benthamiana* leaves. After infiltration, plants were incubated for 48–72 h in the dark conditions at 25 °C. GFP fluorescence was detected using a confocal laser scanning microscope (Leica TCS SP5, Leica Microsystems, Wetzlar, Germany). The GFP signal from 35S::GFP was used as the control.

### 4.9. Yeast Two-Hybrid Assay

The CDS of *PtoWOX1* was amplified and cloned into the pGBKT7 (BD) vector, while the CDS of PtoYAB was cloned into the pGADT7 (AD) vector. The recombinant plasmid was co-transformed into the yeast strain *Saccharomyces cerevisiae* AH109 following the manufacturer’s protocol (Clontech, Mountain View, CA, USA). Transformed yeast cells were selected on SD/-Leu/-Trp medium, and then transferred to SD/-Leu/-Trp/-His/-Ade medium supplemented with X-α-gal for interaction screening. The pGBKT7-p53 and pGADT7-T vectors were used as positive controls, and pGBKT7-lam and pGADT7-T vectors were used as negative controls.

### 4.10. Statistical Analysis

All quantitative data are presented as means ± standard deviation (SD) from at least three independent biological replicates (*n* = 3). Statistical significance was determined using Student’s *t*-test (two-tailed) for pairwise comparisons. Differences were considered statistically significant at *p* < 0.05. Statistical analyses were performed using GraphPad Prism 10.0 (GraphPad Software, San Diego, CA, USA).

## Data Availability

The original contributions presented in this study are included in the article/Supplementary Material. Further inquiries can be directed to the corresponding author.

## Supplementary Materials

The following supporting information can be downloaded at: https://www.mdpi.com/article/10.3390/plants14142138/s1, Figure S1. Subcellular Localization of PtoWOX1A and PtoWOX1B in *P. tomentosa*. The empty GFP vector was used as a negative control. From left to right: green fluorescence, bright field, and merged. Scale bar = 50 μm. Figure S2. Identification of *PtoWOX1* Over-expression Lines in *P. tomentosa*. (A,B) Expression analysis of *PtoWOX1A* (A) and *PtoWOX1B* (B) in *PtoWOX1* over-expressing lines. The poplar *UBQ* was used as the internal reference gene. Data are presented as means ± standard deviation (SD), *n* = 3 biological replicates. Student’s *t*-test: *p* < 0.01 (**). (C) Phenotypes of *PtoWOX1A*-OE and *PtoWOX1B*-OE transgenic plants after two weeks of soil cultivation. Scale bar = 1 cm. Figure S3. Identification of *PtoWOX1* Knockout Lines in *P. tomentosa*. (A) Schematic diagram of CRISPR/Cas9 target site design for *PtoWOX1* gene knockout. (B) Genomic identification of *PtoWOX1* knockout lines (*PtoWOX1*-KO). (C) Phenotypes of *PtoWOX1*-KO plants after two weeks of soil cultivation. Scale bar = 1 cm. Figure S4. Longitudinal Sections of Leaf in *PtoWOX1*-KO (Line 2) Transgenic Lines. (A) Cellular morphology of longitudinal leaf sections in *PtoWOX1* 1-KO (Line 2) transgenic lines. Scale bar = 50 µm. V: vein, Pc: parenchyma cells, Cc: collenchyma cells. (B) Cellular morphology of leaf midvein. Scale bar = 20 µm. Xy: xylem; Ph: phloem. Figure S5. Chlorophyll Content in *PtoWOX1* Transgenic Lines of *P. tomentosa*. Figure S6. Interaction between *PtoWOX1* and PtoYAB3/FIL Proteins in *P. tomentosa*. Yeast two-hybrid assay was used to verify the interaction between *PtoWOX1* and PtoYAB3/FIL. The AD-*PtoWOX1* construct was used as the prey fusion protein, and the BD-*PtoYAB3*/FIL construct was used as the bait. The prey and bait constructs were co-transformed into *Saccharomyces cerevisiae* strain AH109. Co-transformed yeast cells were first grown on double dropout medium (SD/-Trp-Leu), and then transferred to quadruple dropout medium (SD/-Trp-Leu-His-Ade) containing X-α-Gal for interaction verification. Table S1. Sequences of primers used in this study.
