# Supplementary material for: Functional Characterization of PtoWOX1 in Regulating Leaf Morphogenesis and Photosynthesis in Populus tomentosa
Source: Plants (Basel). 2025 Jul 10;14(14):2138. doi: 10.3390/plants14142138 (PMC12299494; doi:10.3390/plants14142138)
Supplement: Supplementary file 1 [file plants-14-02138-s001.zip › plants-3679452-supplementary.pdf]

SUPPLEMENTAL FIGURES

Functional Characterization of *PtoWOX1* in Regulating Leaf Morphogenesis and Photosynthetic in *Populus tomentosa*

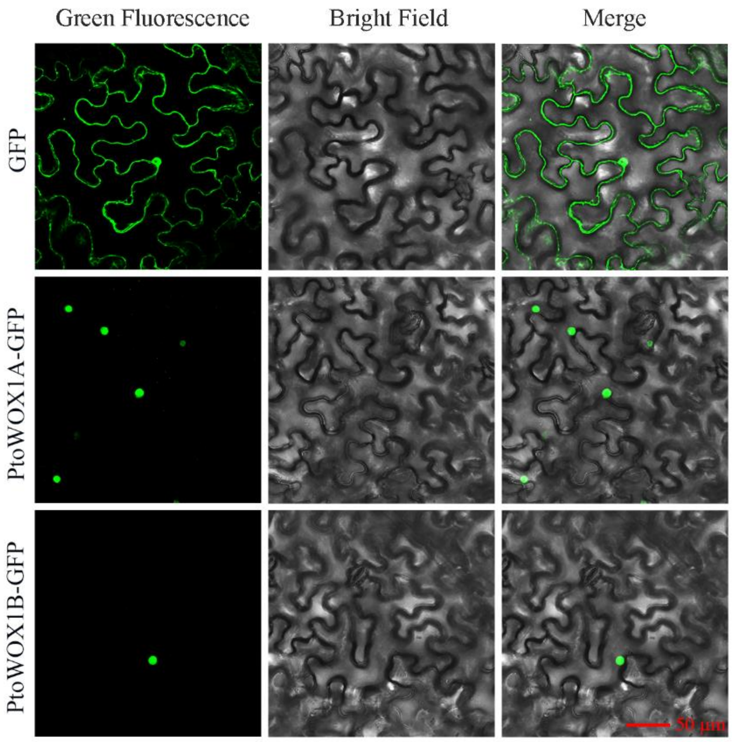

Figure S1. Subcellular Localization of PtoWOX1A and PtoWOX1B in *P. tomentosa*.

The empty GFP vector was used as a negative control. From left to right: green fluorescence, bright field, and merged. Scale bar = 50 μm.

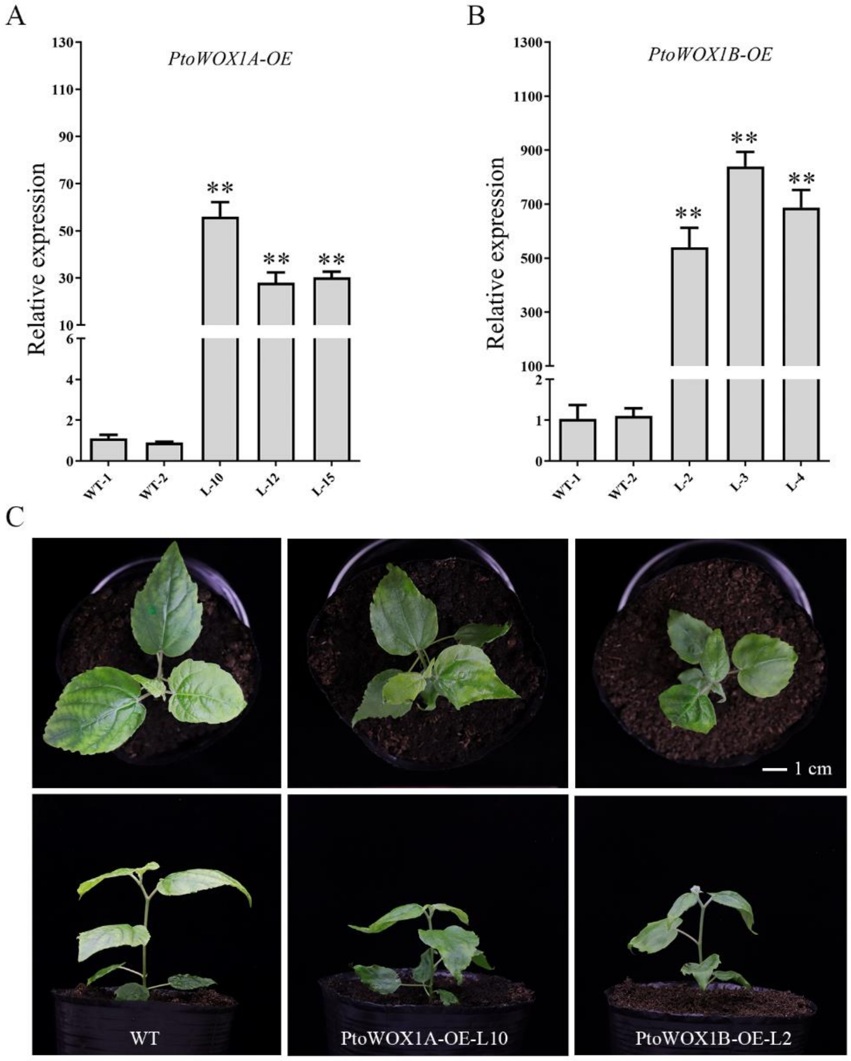

Figure S2. Identification of *PtoWOX1* Over-expression Lines in *P. tomentosa*.

(A-B) Expression analysis of *PtoWOX1A* (A) and *PtoWOX1B* (B) in *PtoWOX1* over-expressing lines. The poplar Ubiquitin gene (UBQ) was used as the internal reference gene. Data are presented as means ± standard deviation (SD), n = 3 biological replicates. Student's t-test: $P < 0.05$ (*), $P < 0.01$ (**).

(C) Phenotypes of PtoWOX1A-OE and PtoWOX1B-OE transgenic plants after two weeks of soil cultivation. Scale bar = 1 cm.

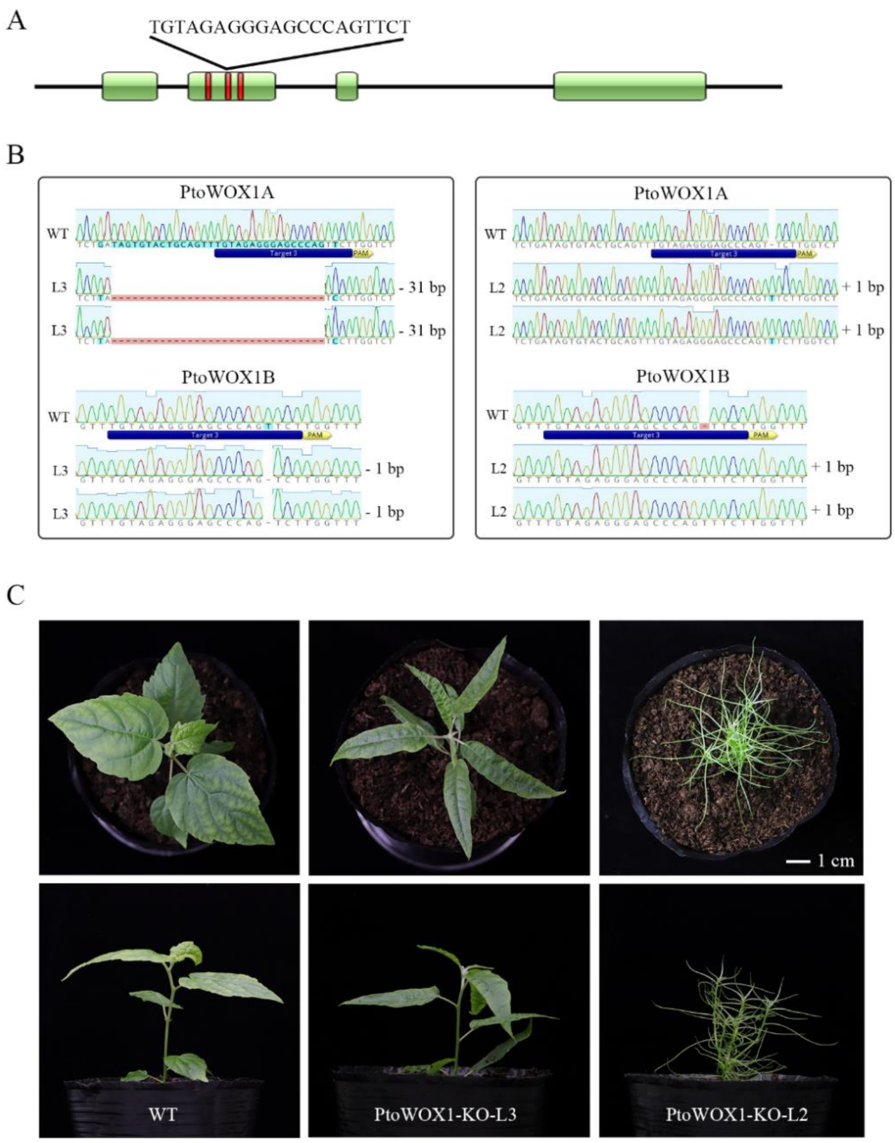

Figure S3. Identification of *PtoWOX1* Knockout Lines in *P. tomentosa*.

(A) Schematic diagram of CRISPR/Cas9 target site design for *PtoWOX1* gene knockout.

(B) Genomic identification of *PtoWOX1* knockout lines (PtoWOX1-KO).

(C) Phenotypes of PtoWOX1-KO plants after two weeks of soil cultivation. Scale bar = 1 cm.

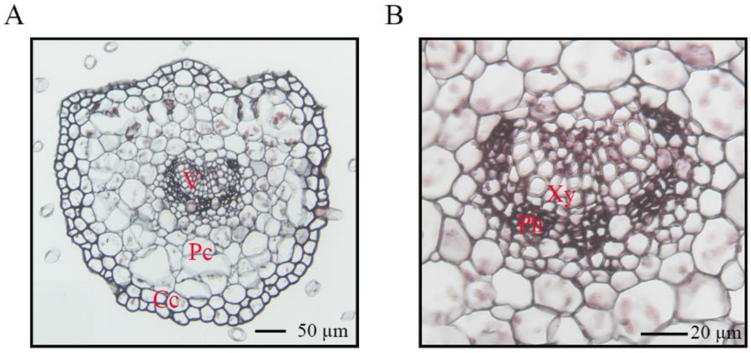

Figure S4. Longitudinal Sections of Leaf in *PtoWOX1-KO* (Line 1) Transgenic Lines.

(A) Cellular morphology of longitudinal leaf sections in *PtoWOX1* 1-KO (Line 2) transgenic lines. Scale bar = 50 μm. V: vein, Pc: parenchyma cells, Cc: collenchyma cells.

(B) Cellular morphology of leaf midvein. Scale bar = 20 μm. Xy: xylem; Ph: phloem

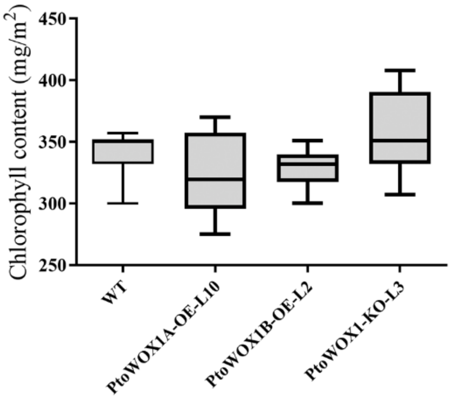

Figure S5. Chlorophyll Content in *PtoWOX1* Transgenic Lines of *P. tomentosa*.

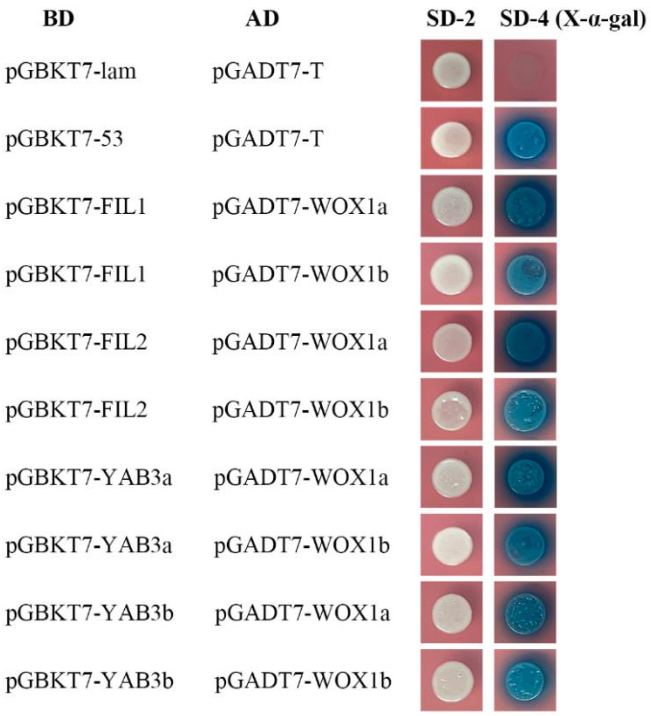

Figure S6. Interaction between *PtoWOX1* and PtoYAB3/FIL Proteins in *P. tomentosa*.

Yeast two-hybrid assay was used to verify the interaction between *PtoWOX1* and PtoYAB3/FIL. The AD-*PtoWOX1* construct was used as the prey fusion protein, and the BD-*PtoYAB3*/FIL construct was used as the bait. The prey and bait constructs were co-transformed into *Saccharomyces cerevisiae* strain AH109. Co-transformed yeast cells were first grown on double dropout medium (SD/-Trp-Leu), and then transferred to quadruple dropout medium (SD/-Trp-Leu-His-Ade) containing X-α-Gal for interaction verification.

Table S1. Sequences of primers used in this study

Primer Name	Primer Sequence（5'-3'）
PtoWOX1A-F	AATGTGGATGATGGGTTACAAT
PtoWOX1A-R	TTAGATCCTCAGTGGAAGGAACT
PtoWOX1B-F	AATGTGGATGATGGGTTATAATG
PtoWOX1B-R	TTAGTGCTTCAGTGGAAGGAAC
PtoWOX1C-F	CATGTGGATGATAAATGGTGG
PtoWOX1C-R	TCAGTTCTTCAGAGGAAGAAACTC
Q-W1A-F	CAAGTGCGTCAACACAAGGAG
Q-W1A-R	TTCGTCATTGCCATCTCTGCA
Q-W1B-F	GGAGCAATGGCAGAGTACTGT
Q-W1B-R	CTTAATGAGCTTCGCTGCTGC
Q-W1C-F	TCAAGCCTTGAAGAGCACAGT
Q-W1C-R	GCTTGGGGTGAAGGTCGTATT
Q-AS2-F	TATGGCTGCGTTGGAGTCATT
Q-AS2-R	GGATGGTGGTGATGATGGGTT
Q-PHB-F	CATTTGTGAACTGCAGGAGGC
Q-PHB-R	TCGCATTGACGTTGAAGGGTA
Q-KAN4-F	AGCCCTTTGCAGTGATAGGAG
Q-KAN4-R	GGTTGGTGGTGGTGGTGATTA
Q-YAB3B-F	CCAATCTGCTCCCTGTCAACA
Q-YAB3B-R	TGATCAGCCATTCCTCGAACC
Q-UBQ-F	GTTGATTTTTGCTGGGAAGC
Q-UBQ-R	GATCTTGGCCTTCACGTTGT
HYG-F	TTCTGCGGGCGATTTGTG
HYG-R	CGTGCTTTCAGCTTCGATGTA
NPT-F	CTATTCGGCTATGACTGGGC
NPT-R	AATATCACGGGTAGCCAACG
pCXSN-F	ATCTCAAGCAATCAAGCATTC
pCXSN-R	AAGACCGGCAACAGGATT
